# New Evidence for the Role of Ceramide in the Development of Hepatic Insulin Resistance

**DOI:** 10.1371/journal.pone.0116858

**Published:** 2015-01-30

**Authors:** Karolina Konstantynowicz-Nowicka, Ewa Harasim, Marcin Baranowski, Adrian Chabowski

**Affiliations:** Department of Physiology, Medical University of Bialystok, Białystok, Poland; Medical University of South Carolina, UNITED STATES

## Abstract

**Aim:**

There are few and contradictory data on the role of excessive accumulation of intracellular sphingolipids, particularly ceramides, in the development of hepatic insulin resistance. In our study we assessed accumulated sphingolipid fractions and clarify the mechanisms of hepatic insulin resistance development as well as involvement of fatty acid and ceramide transporters in this process.

**Methods:**

In culture of primary rat hepatocytes, exposed to high concentration of palmitic acid (0.75mM) during short and prolonged incubation, high performance liquid chromatography was used to assess intra- and extracellular sphingolipid fractions content. Degree of palmitate-induced insulin resistance was estimated by measuring changes in phosphorylation of insulin pathway proteins by western blotting as well as changes in expression of different type of transporters.

**Results:**

In our study short and prolonged exposure of primary hepatocytes to palmitic acid resulted in increased intracellular accumulation of ceramide which inhibited insulin signaling pathway. We observed a significant increase in the expression of fatty-acid transport protein (FATP2) and ceramide transfer protein (CERT) what is consistent with enhanced intracellular ceramide content. The content of extracellular ceramide was increased nearly threefold after short and twofold after long incubation period. Expression of microsomal triglyceride transfer protein (MTP) and ATP-binding cassette transporter (ABCA1) was increased significantly mainly after short palmitate incubation.

**Conclusion:**

Our data showed that increase in intarcellular ceramide content contributes to the development of hepatic insulin resistance. We suggest pivotal role of transporters in facilitating fatty acid influx (FATP2), accumulation of ceramides (CERT) and export to the media (MTP and ABCA1).

## Introduction

The incidence of obesity and type 2 diabetes mellitus is reaching epidemic proportions worldwide. Common association of insulin resistance with these two diseases has caused a rapid expanse of research investigating the link between lipid metabolism and the pathogenesis of insulin resistance [1]. In obesity, fatty acid storage capacity of adipocytes is exceeded and lipids accumulate in other tissues such as muscles and liver [2,3]. Numerous studies indicate that exposing cultured myotubes to high concentration of palmitic acid, the most abundant saturated fatty acid in the circulation, leads to excessive accumulation of biologically active sphingolipids such as ceramides, which can induce insulin resistance by inhibition of Akt/PKB phosphorylation [4,5].

As far most data concerning the relationship of sphingolipids (especially ceramides) and insulin resistance are based on skeletal muscle studies and the molecular mechanisms that underlie hepatic insulin resistance are still not clear [6]. Although, the molecular mechanisms of ceramide-induced insulin resistance are complex and not completely understood, particularly in hepatocytes, there is emerging evidence for fatty acid transport involvement in increased fatty acids influx followed by excessive free fatty acid accumulation [7–9]. It is now well acknowledged that fatty acid uptake is facilitated by a number of membrane proteins such as fatty acid translocase (FAT/CD36), plasma membrane-associated fatty acid-binding proteins (FABPpm) and fatty-acid transport proteins (FATP2). Recent studies have shown the presence of these transporters also in hepatocytes, but the precise mechanism of fatty acid transporters action and their role in liver remains unclear [10].

**Figure 1:**
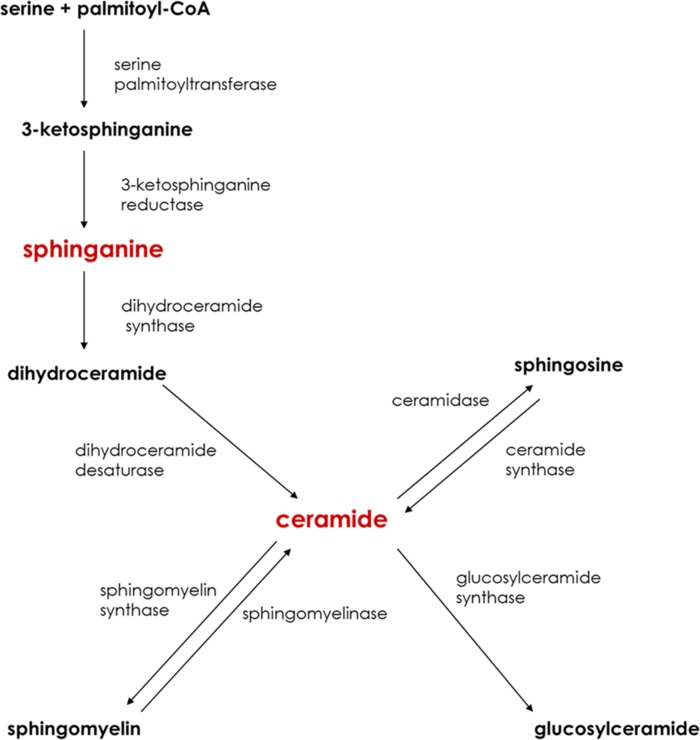
Major pathways of sphingolipid metabolism. Sphingolipids indicated in red color are putative contributors in the development of hepatic insulin resistance.

Delivery of saturated fatty acids, such as palmitate, into hepatocytes is crucial to the *de novo* ceramide synthesis (Fig. 1). Ceramide can be also generated from the hydrolysis of the sphingomyelin and once generated, ceramide is the common precursor of other sphingolipids or can be transported outside of the cell (Fig. 1) [11,12]. Among the proteins involved in lipid secretion from hepatocytes, the most studied are microsomal triglyceride transfer protein (MTP) and ATP-binding cassette transporter (ABCA1). It is suspected that MTP is mainly involved in transporting ceramide between cell compartments and facilitating sphingolipid incorporation into lipoproteins and ABCA1 is thought to regulate sphingolipids efflux from hepatocytes. However the role of these transporters in ceramide export and in the development of hepatic insulin resistance is not known. The synthesis of sphingolipid intermediates depends on the intracellular transport efficiency between cell organelles [13]. CERT is a protein that is responsible for the transport of ceramide from the endoplasmic reticulum to the Golgi apparatus where ceramide is converted to sphingomyelin [14].

There are few and somewhat contradictory data on the role of ceramide in the development of hepatic insulin resistance. Longato L. et all found that ceramide as a main bioactive lipid fraction can interfere with insulin signaling pathway. Accumulation of this sphingolipid in liver, induced by high fat diet, caused hepatic insulin resistance [15]. In contrast, a recent report from Galbo T. et all showed that accumulation of diacylglycerols, but not ceramides after diet rich in saturated and unsaturated fatty acids impair insulin signaling in hepatocytes [16]. Interestingly most data concerning ceramide and insulin resistance are based on skeletal muscle and little is known about mechanism involved in the development of hepatic insulin resistance and precise role of ceramides in this process. Therefore, in this study, we challenged the elucidation of the relationship between hepatic insulin resistance and accumulation of sphingolipid fractions during short and prolonged exposure to palmitic acid. Furthermore, we clarified why some researchers received different results because we analyzed the amount of ceramide transported to the media. Our experiments described herein also were focused on the assessment of proteins involved in fatty acid and sphingolipid transport, what shed more light on possible molecular mechanisms related to sphingolipid accumulation and the development of insulin resistance in liver.

## Materials and Methods

### Primary rat hepatocytes isolation and culture

This study was approved by the ethics committee on animal care at the Medical University of Bialystok. Male Wistar rats (200–250 g) were housed in approved animal holding facilities (at 22°C±2), on a reverse light-dark cycle, with unrestricted access to water and standard laboratory rat chow. Primary rat hepatocytes were isolated from rat liver by the two-step EDTA and collagenase perfusion according to method of Seglen [17]. Briefly, after anesthetizing the rat by intraperitoneal injection of pentobarbital in a dose of 80 mg kg^−1^ of body weight, liver was perfused through the hepatic portal vein with Hank’s Balanced Salt Solution (HBSS, Immuniq, Zory, Poland) containing EDTA (0.05 mM). Next, the liver was treated with HBSS containing 0.05% collagenase (Sigma-Aldrich, St. Louis, MO) until the disruption of intercellular junctions and destruction of the liver structure. Thereafter, the liver was removed and gently minced, obtained cells were dispersed in medium (DMEM, Immuniq, Zory, Poland) containing 10% fetal bovine serum, 1% antibiotic/antimycotic, 1% HEPES (Sigma-Aldrich, St. Louis, MO). The resulting solution containing mixed cells and debris was filtered through 100 μm filter. Subsequently, the remaining filtrate was centrifuged at 1000 g for 3 minutes at 4°C and washed three times with DMEM and the cells were seeded in 12 well collagen-coated plates. The cells were maintained in DMEM (Immuniq, Zory, Poland) supplemented with 10% fetal bovine serum (FBS, Polgen) and 1% antibiotic/antimycotic (penicillin/streptomycin, Immuniq, Zory, Poland) for 24 h. Cells were cultured at 37°C in a humidified atmosphere (5% CO_2_), and after 24h the cells were washed twice with PBS (Immuniq, Zory, Poland) and the morphology and viability of the attached primary hepatocytes was assessed in Bürker chamber using Trypan blue (Sigma-Aldrich, St. Louis, MO) staining during all incubation periods. Each experiment was always carried out on 10^6^ cells per well, in which the percentage of living cells was above 85%.

### Fatty acid treatment

The treatment with palmitic acid (PA) was conducted on serum-starved cells as previously described [18]. Before the addition of palmitate to the medium, it was conjugated with fatty acid-free bovine serum albumin (BSA, Sigma-Aldrich, St. Louis, MO). Briefly, palmitate stock solution was prepared by dissolving PA in a mixture of absolute ethanol and 1 M NaOH, heating to 70°C and conjugating with 10% BSA. Next, stock solution was diluted in serum free-DMEM, containing 10mM Hepes. Next the cells were incubated in the presence or absence of the palmitic acid at the concentration of 0.75 mM for 16h and 40h. At the end of each experimental set cells morphology as well as viability was assessed (i.e. Trypan blue staining). In selected sets (as indicated in Figures) the hepatocytes were chased by 100nM insulin (NovoRapid, Novo Nordisk, Ontario, Canada) for 15 min at 37°C then washed three times with PBS, harvested and homogenized in ice cold RIPA lysis buffer containing protease and phosphatase inhibitors (Roche Diagnostics GmbH, Mannheim, Germany). Other sets of palmitate induced insulin resistant hepatocytes were transferred for further analysis of glycogen synthesis or sphingolipid content.

### Glycogen synthesis

Glycogen synthesis was determined by estimating insulin stimulated D-[U-^14^C]glucose (Perkin Elmer, Waltham, USA) incorporation [19]. Glycogen synthesis assay was performed in 6-well collagen coated plates. Cells were washed with PBS and after the palmitate-induced insulin resistance, incubated in DMEM containing Hepes (25 mM) and D-[U-^14^C]glucose (18.5 MBq/l) with or without insulin (100 nM) for 1 h at 37°C. Next, the reaction was terminated by three rapid washes with ice cold PBS and cells were lysed in 1 M KOH. Cell lysates were heated to 90°C for 15 min and 10 μl of samples were taken for protein quantification. In order to precipitate synthetized glycogen saturated solution of Na_2_SO_4_ and absolute ethanol were added. Samples were incubated 15 min at −80°C, centrifuged at 20,000 × g for 10 min at 4°C and then pellets were washed with water, resuspended in ice cold 70% ethanol and centrifuged again. At this point, obtained pellets in water were transferred to scintillation vials and mixed with 5 ml of scintillation cocktail. Incorporation of D-[U-^14^C]glucose was measured by scintillation counting on a Beckman Scintillation counter.

### Sphingolipid analyses

The content of ceramide, sphingosine and sphinganine was determined as previously described in detail [20]. Briefly, hepatocytes and medium were homogenized by ulatrasonication and lipids were extracted in the presence of internal standard (10 pmol of C17-sphingosine, Avanti Polar Lipids). An aliquot of the lipid extract was transferred to a fresh tube with pre-added 40pmol of N-palmitoyl-D-erythro-sphingosine (C17 base) (a kind of gift from Dr Z. Szulc, Medical University of South Carolina) as an internal standard, and then subjected to alkaline hydrolysis to deacylate ceramide to sphingosine. Free sphinganine, as well as sphingosine released from ceramide were then converted to their o-phthalaldehyde derivatives and analyzed using a HPLC system equipped with a fluorescence detector and C18 reversed-phase column (Varian Inc. OmniSpher 5, 4.6×150mm). The isocratic eluent composition of acetonitrile (Merck): water (9:1, v/v) and a flow rate of 1 ml/min were used. Prior to the sphingolipid analysis, the protein content was measured in all samples with bovine serum albumin (Sigma-Aldrich, St. Louis, MO) as a standard.

### Immunoblotting

Routine western blotting procedures were used to detect the expression of proteins and their phosphorylated forms directly involved in insulin signaling pathway [Akt, pAkt (Ser473), GSK, pGSK (Ser9), IRS2, pIRS2 (Ser731) all purchased from Cell Signaling Technology, USA] and the expression of sphingolipid pathway enzymes [SPTLC2, SPTLC1 (Santa Cruz Biotech, CA, USA) and LASS6 (Sigma-Aldrich, St. Louis, MO)]. Furthermore, fatty acid [FAT/CD36 (Novus Biologicals, Cambridge), FABPpm, (a gift from Arend Bonen), FATP2 (Santa Cruz Biotech, CA, USA)] and ceramide transfer proteins [CERT (Abcam, EU), MTP (Santa Cruz Biotech, CA, USA), ABCA1 (Thermo Scientific, USA)] as well as protein involved in apoptotic pathway (Bcl10, Cell Signaling Technology, USA) were measured as described previously [21]. In brief, before the Western Blot procedures hepatocytes were homogenized in RIPA buffer containing protease and phosphatase inhibitors (Roche Diagnostics GmbH, Mannheim, Germany). Total protein concentration was determined using bicinchonic acid method (BCA) with BSA as a standard. Next, cell lysates were reconstituted in Laemmli buffer, separated by 10% sodium dodecyl sulfate-polyacrylamide gel electrophoresis (SDS-PAGE) using a Bio-Rad kit and transferred onto nitrocellulose membranes. After blocking with 5% nonfat milk in phosphate buffered saline the membranes were immunoblotted with primary antibodies of interest and incubated with appropriate horseradish peroxidase—labeled secondary antibody (Santa Cruz Biotechnology, CA). Equal protein concentration loading were controlled by Ponceau S staining. After adding a suitable substrate for horseradish peroxidase (Thermo Scientific, Rockford, USA) protein bands were quantified densitometrically using a ChemiDoc visualization system EQ (Bio Rad, Warsaw, Poland). The protein expression was standardized to β-actin (Sigma-Aldrich, St. Louis, MO) expression, and the control group was set to 100%. The results are shown graphically as a percentage change (%) in relation to the control group.

### Statistical analysis

Data are expressed as mean values ± SEM based on six independent determinations. Statistical difference between groups was tested with analyses of variance and appropriate post hoc tests, or with a Student t-test using Statistica version 10 (StatSoft, Krakow, Poland). Results were considered statistically significant at P ≤ 0.05.

## Results

### Effects of 16h and 40h incubation of primary hepatocytes with PA on intracellular synthesis and accumulation of sphingolipids (SFA, CER, SFO)

**Figure 2:**
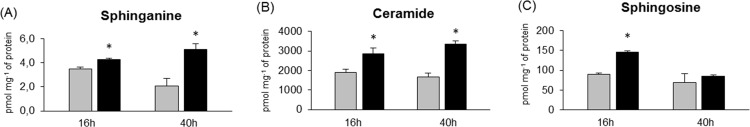
Intracellular content of sphinganine (A), ceramide (B) and sphingosine (C) in primary rat hepatocytes. Cells were incubated in high glucose DMEM culture medium without (Control group) or with palmitate (0.75 mM) (Palmitate group) for 16h and 40h. Thereafter primary hepatocytes from each experimental group were prepared as it was described previously in Materials and Methods. Protein concentration was measured by BCA method and sphingolipids were determined by HPLC (see Materials and Methods). The data are expressed as the mean ± SEM and are based on six independent determinations (n = 6). *P<0.05 significant difference: Control group vs. Palmitate group. Grey bars are referred as control groups and black bars are referred as palmitate groups.

**Figure 3:**
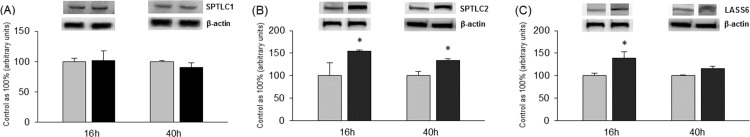
Expression of enzymes involved in hepatic sphingolipid metabolism i.e. SPTLC1 (A), SPTLC2 (B) and LASS6 (C) in primary rat hepatocytes. Cells were incubated in high glucose DMEM culture medium without (Control group) or with palmitate (0.75 mM) (Palmitate group) for 16h and 40h. Thereafter primary hepatocytes from each experimental group were prepared as it was described previously in Materials and Methods. Protein concentration was measured by BCA method. Expression of LASS6, SPTLC1 and SPTLC2 was analyzed by western blotting. The data are expressed as the mean ± SEM and are based on six independent determinations (n = 6). *P<0.05 significant difference: Control group vs. Palmitate group. Grey bars are referred as control groups and black bars are referred as palmitate groups.

Substantial changes were observed in sphingolipid metabolism in response of primary hepatocytes to PA treatment during short and long incubation period. There was a considerable increase in CER content in examined groups of primary hepatocytes incubated with PA for 16h (PA: +49.8%, P<0.05, Fig. 2B) and 40h (PA: +99.1%, P<0.05, Fig. 2B). High availability of palmitic acid in culture medium intensified de novo synthesis of CER due to concomitant increase in content of SFA during both incubations times. We noticed a significant increase in intracellular SFA fraction after short (PA: +22.6%, P<0.05, Fig. 2A) and long (PA: +144.4%, P<0.05, Fig. 2A) incubation time in PA-treated groups compared with control groups. Moreover sphingosine content in primary hepatocytes increased considerably after short PA treatment (PA: +63.8%, P<0.05, Fig. 2C). Also incubation of primary hepatocytes with PA caused a substantial changes in intrahepatocellular expression of key enzymes involved in sphingolipid metabolism. Primary hepatocytes incubated for 16h with PA revealed a considerable increase in expression of both enzymes i.e. SPTLC2 (PA: +54.3%, P<0.05, Fig. 3B) and LASS6 (PA: +39.5%, P<0.05, Fig. 3C) compared with control group with exception of SPTLC1 (Fig. 3A). Moreover, we also observed a tendency towards elevation of expression of these proteins in primary hepatocytes after long term incubation (SPTLC2, PA: +34%, P<0.05, Fig. 3B; LASS6, PA: +15.44%, P>0.05, Fig. 3C).

### Effects of 16h and 40h incubation of primary hepatocytes with PA on expression of insulin signaling pathways proteins (IRS2, pIRS2, Akt, pAkt, GSK, pGSK)

**Figure 4:**
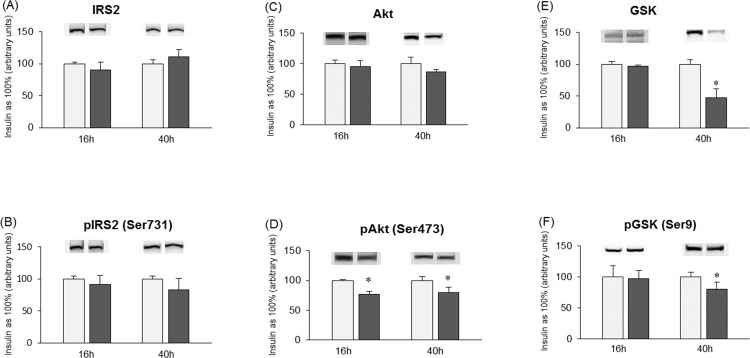
Expression of insulin signaling pathways proteins IRS2 (A), pIRS2 (B), Akt (C), pAkt (D), GSK (E) and pGSK (F) in primary rat hepatocytes. Cells were incubated in high glucose DMEM culture medium without (Insulin group) or with addition of palmitate (0.75 mM) (Palmitate + Insulin group) for 16h and 40h. Furthermore, after finishing each incubation period, all experimental groups were treated with insulin (100 nM) for 15 minutes. Thereafter primary hepatocytes from each experimental group were prepared as it was described previously in Materials and Methods. Protein concentration was measured by BCA method. Expression of these proteins was analyzed by western blotting (see Materials and Methods). The data are expressed as the mean ± SEM and are based on six independent determinations (n = 6). *P<0.05 significant difference: Insulin group vs. Palmitate + Insulin group. Light grey bars are referred as insulin groups and dark grey bars are referred as palmitate + insulin groups.

**Figure 5:**
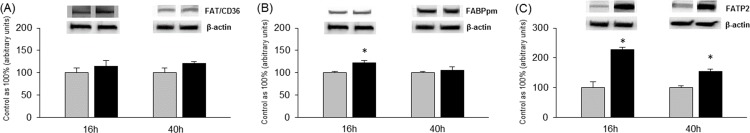
Expression of proteins involved in plasmalemmal transport of fatty acids i.e. FAT/CD36 (A), FABPpm (B) and FATP2 (C) in primary rat hepatocytes. Cells were incubated in high glucose DMEM culture medium without (Control group) or with palmitate (0.75 mM) (Palmitate group) for 16h and 40h. Thereafter primary hepatocytes from each experimental group were prepared as it was described previously in Materials and Methods. Protein concentration was measured by BCA method. Total expression of these proteins were analyzed by western blotting. The data are expressed as the mean ± SEM and are based on six independent determinations (n = 6). *P<0.05 significant difference: Control group vs. Palmitate group. Grey bars are referred as control groups and black bars are referred as palmitate groups.

The expression of a key proteins involved in hepatic insulin signaling pathways in primary rat hepatocytes was affected during short (16h) and long (40h) term incubations with palmitate. Palmitic acid induced reduction in response of primary hepatocytes to insulin by inhibiting insulin-stimulated phosphorylation of Akt (16h, PA+INS: −23.3%, P<0.05; 40h, PA+INS: −20.52%, P<0.05, Fig. 4D) and GSK (16h, PA+INS: −2.6%, P>0.05; 40h, PA+INS: −20%, P<0.05, Fig. 5F). Total expression of Akt (16h, PA+INS: −4.7%, P>0.05; 40h, PA+INS: −13.5%, P>0.05, Fig. 4C) and GSK (16h, PA+INS: −3.1%, P>0.05, Fig. 4E) was not significantly changed by palmitic acid except for decline in GSK expression after 40h (PA+INS: −52.3%, P<0.05, Fig. 4E) incubation compared with insulin treated cells. Also we did not notice any considerable alternations in total expression of IRS2 (16h, PA+INS: −9.4%, P>0.05; 40h, PA+INS: +11.2%, P>0.05, Fig. 4A) after palmitate treatment. Insulin dependent phosphorylation of IRS2 was slightly reduced, especially after long term palmitate treatment (16h, PA+INS: −8.5%, P>0.05; 40h, PA+INS: −17.6%, P>0.05, Fig. 4B).

### Effects of 16h and 40h incubation of primary hepatocytes with PA on total expression of fatty acid transporters (FAT/CD36, FABPpm, FATP2)

Expression of the examined fatty acid transporters, especially FATP2, was changed in primary hepatocytes in response to high concentration of PA for 16h and 40h. The presence of PA in culture medium for 16h and 40h caused a substantial increase in FATP2 expression compared with control group (16h, PA: +127.2%, P<0.05, Fig. 5C; 40h, PA: +54.4%, P<0.05, Fig. 5C). Moreover at the end of both incubation periods there was a slight rise of expression of FAT/CD36 (16h, PA: +14%, P>0.05; 40h, PA: +20.7%, P>0.05, Fig. 5A) and FABPpm (16h, PA: +21.8%, P<0.05; 40h, PA:+5.2%, P>0.05, Fig. 5B) in all PA-treated groups of primary hepatocytes, but the increase was relatively lower compared with FATP2.

### Effects of 16h and 40h incubation of primary hepatocytes with PA on intracellular transport and extracellular secretion of ceramide

**Figure 6:**
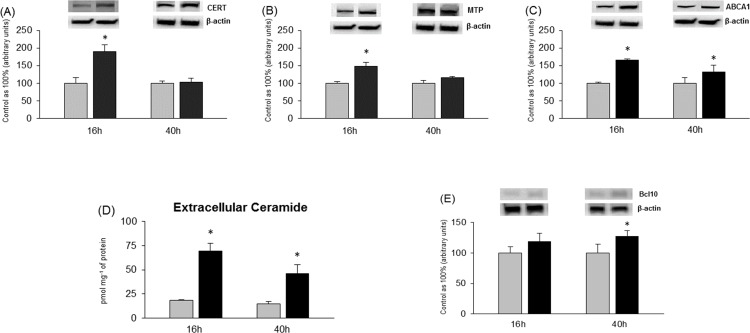
Expression of proteins involved in intracellular and extracellular transport of sphingolipids i.e. CERT (A), MTP (B) and ABCA1 (C) in primary hepatocytes. Extracellular content of ceramide (CER) (D) in incubation media of primary hepatocytes. Expression of protein involved in apoptotic pathway i.e. Bcl10 (E). Isolated hepatocytes were incubated in high glucose DMEM culture medium without (Control group) or with palmitate (0.75 mM) for 16h and 40h. Thereafter primary hepatocytes from each experimental group were prepared as it was described previously in Materials and Methods. Protein concentration was measured by BCA method. Expression of these proteins were analyzed by western blotting. At the end of experiments incubation media from all groups of primary hepatocytes were separated from the cells. Content of secreted CER into the incubation medium was determined by HPLC (see Materials and Methods). The data are expressed as the mean ± SEM and are based on six independent determinations (n = 6). *P<0.05 significant difference: control group vs. palmitate group. Grey bars are referred as control groups and black bars are referred as palmitate groups.

We also observed a significant increase in expression of CERT in primary hepatocytes especially incubated with PA for 16h (PA: +68.4%, P<0.05, Fig. 6A). Expression of CERT after 40h incubation with PA was still changed but not substantially compared with control group (PA: +14.9%, P>0.05, Fig. 6A). Similarly to CERT expression, there was a substantial rise in expression of MTP protein (16h, PA: +48.4%, P<0.05; 40h, PA: +16.1%, P>0.05, Fig. 6B) after incubation with PA in primary rat hepatocytes. Interestingly, increased expression of the transporters (CERT, MTP) after 16h incubation with PA preceded induction of apoptosis occurred after 40h incubation period (Fig. 6E). Total expression of ABCA1 was also considerably elevated in PA-treated groups compared with control groups after short and long incubation period (16h, PA: +66%, P<0.05; 40h, PA: +31.1%, P<0.05, Fig. 6C). Probably, increased expression of proteins responsible for secretion of sphingolipids resulted in significant accumulation of ceramide in culture media. Extracellular content of CER was substantially elevated in all groups incubated with PA compared with control groups. The increase was nearly threefold in primary hepatocytes incubated for 16h (PA: +276.1%; P<0.05, Fig. 6D) and twofold after 40h of experiment (PA: +208.3%, P<0.05, Fig. 6D) in comparison with control group.

## Discussion

The goal of our study was to elucidate whether the increased availability of fatty acids (PA) causes ceramide accumulation in hepatocytes and leads to the development of insulin resistance. We also investigated the mechanism of fatty acid and sphingolipid transport crucial for excessive accumulation of intra- and extracellular lipids, what may explain the discrepancies observed in various studies (that have been so far presented).

The data presented herein showed that short (16h) and prolonged (40h) incubation with palmitate increased intracellular sphingolipids concentration namely ceramide and sphinganine. This is in agreement with other studies showing that accumulation of ceramide specifically in skeletal muscles due to increased palmitate availability, is able to induce insulin resistance [4]. In our study, in primary rat hepatocytes we observed that palmitate increased intracellular ceramide concentrations, impaired insulin action by inhibiting protein kinase B and glycogen synthase kinase dephosphorylation. As a result, glycogen synthase kinase inhibition impaired glycogen synthesis (data not shown) what is commonly considered as an indicator of hepatic insulin resistance. Our previous studies conducted in HepG2 cells indicated also that palmitate but not oleate can inhibit phosphorylation of insulin receptor and insulin receptor substrate (IRS2) [22]. Herein, in primary hepatocytes, we showed that prolonged palmitate exposure (40h) resulted in notable impairment of insulin signaling downstream proteins (pAkt and pGSK).

However not all studies show that ceramide is the main accumulated lipid fraction that may induce insulin resistance in liver [16,23]. It is suspected that also other sphingolipid fractions such as glycosylated ceramide may contribute to the development of hepatic insulin resistance [24]. Based on that our aim was to understand the role of ceramide in insulin resistant states and assess the potential routes by which hepatocytes can get rid of excess ceramide.

Ceramide serves as a hub in sphingolipid metabolism [25], thus we assessed ceramide de novo pathway by measuring the expression of two enzymes serine palmitoyltransferase (SPTLC2) and dihydroceramide synthase (LASS6). As we suspected the expression of these two rate-limiting enzymes was elevated what is in accordance with increased sphinganine and ceramide content in hepatocytes. We did not include investigation of other sphingolipid routes such as sphingomyelin hydrolysis in the present study. We paid more attention to factors that affect sphingolipid de novo pathway. Ceramide and other sphingolipids from de novo pathway may be also generated from sphingosine through salvage pathway [26]. However in our study this recycling pathway is probably not impacted on because we observed increased sphingosine level. We suspect that the cell is protecting itself against already high accumulation of ceramide. As previous studies, conducted in C2C12 myoblasts, indicated the inhibition of enzymes such as SPTLC2 or ceramide synthases resulted in significant decrease in ceramide content, what shows that the main route of ceramide synthesis is de novo pathway [27]. It was of much interest to assess the participation of fatty acid transport proteins in increased ceramide synthesis. The data presented herein demonstrate that fatty acid transport facilitated by transporters is much more effective in short term palmitate incubation than 40h. Taking these data into account, our present findings that fatty acid transport protein (FATP2) expression increased significantly after short and long palmitate incubation suggest that FATP2 may play a major role in fatty acid transport into hepatocytes. Supporting this conclusion are studies revealing that FATP2 knockdown in mice resulted in significant decrease in hepatic fatty acid uptake and total liver triacylglycerols content [28]. There are some findings presenting that fatty acid translocase (FAT/CD36) expression, which is important fatty acid transporter, is increased in patients with nonalcoholic fatty liver disease and CD36 is involved in increased FFA uptake [10]. However we did not observed any significant changes in FAT/CD36 expression after palmitate incubation. One of the most possible explanation of this data is that we measured total FAT/CD36 expression not plasmalemmal as we have described in detail elsewhere [21].

As we indicated above, increased availability of palmitate, resulted not only in excessive accumulation of selected sphingolipids in hepatocytes but also elevated ceramide secretion to the media. There is only one report elucidating that liver cells accumulate and secrete ceramide [29]. The authors of these data have found that hepatocytes detect when their sphingolipid storage capacity is exceeded, and sphingolipids are secreted outside the cell. This extracellular transport may be an important protective mechanism in hepatocytes against lipotoxicity [29] and it precedes cell death. Lipotoxic effects of palmitate include impairements in insulin signalling, induction of insulin resistance, necrosis and apoptosis in different cell types [30] are quite well known and other researchers indicated that palmitate exposure may induce hepatocytes apoptosis [31,32]. It seems likely that hepatic apoptosis parallels or is preceded by increased ceramide secretion since both processes are highly dependent on increased intracellular hepatic ceramide accumulation. It is possible that cell death induced by increased intracellular ceramide accumulation also may be one of the causes of increased ceramide secretion.

This is the first time that the hepatic intra- and extracellular sphingolipid transport and its role in hepatic insulin resistance development have been examined.

In our study we show that ceramide transfer protein (CERT) is involved in accumulation of ceramide and more complex sphingolipids. Since the ceramide metabolism is highly compartmentalized, we observed significant increase in the expression of CERT after short incubation period. We hypothesized that newly synthesized ceramide transported from ER to Golgi is converted to sphingomyelin, which may be further metabolized to other also bioactive molecules or in turn may be hydrolyzed to form ceramide and resulted in intensified ceramide accumulation. However, prolonged incubation with palmitate resulted in only slight increase in CERT expression what may indicate that palmitic acid is possible to cause dysfunction of facilitated by CERT intracellular transport what leads to ceramide accumulation rather than ceramide metabolism. In our study we also found that 16h incubation with palmitic acid brought to significant, consistent with CERT, increase in microsomal triglyceride transfer protein expression. Though MTP is known to be bona fide triglyceride transporter, it also play a relevant role in sphingolipid transport between membranes and forming VLDL particles [33]. Interestingly, longer palmitate incubation period (in comparison with 16h) resulted in decreased ceramide accumulation in media what is coincident with decreased expression of CERT, MTP and ABCA1 at that time. Taking these data into account, we suggest that during 40h incubation hepatocytes protective mechanisms (secretion to the media) are impaired and result in ceramide accumulation in the cells, what is likely to contribute to insulin resistance.

Our results also indicate that MTP and CERT may play an important role in transporting mainly ceramide between cell subfractions and facilitate sphingolipid incorporation into lipoproteins. Moreover MTP together with ATP-binding cassette transporter (ABCA1) facilitates and regulates rate of lipid and sphingolipid efflux from hepatocytes [34]. In line with these statements, we found that increase in ABCA1 expression after 16h as well as 40h incubation is correlated with the rate of ceramide secretion to the media. Overall, these findings suggest that hepatocyte transport of excess sphingolipids out of the cell is highly regulated via different types of transporters, what is supposed to be a liver protective mechanism. Another novelty of our study is that we demonstrated that hepatic insulin resistance develops after long palmitate incubation whereas 16h incubation resulted mostly in intensified transport of lipids into the cell and increased accumulation of ceramide as well as its enhanced export outside the cell. Although, longer palmitate exposure caused that hepatocytes protective mechanism are overwhelmed and as a result fatty acid transport to and outside the cell is decreased, but ceramide accumulation is greatly increased what resulted in defective insulin action. We suspect that increase in extracellular ceramide content may contribute to the development of insulin resistance of other tissues such as skeletal muscles or entire body insulin resistance. However, this issue should be investigated in future studies.

In conclusion, this study demonstrates that short as well as long incubation with palmitate resulted in increased accumulation of ceramide in hepatocytes what caused development of insulin resistance. What is more, we found that in palmitate-treated cells fatty acid transporters expression was increased, mainly FATP2, what suggest that it takes part in fatty acid influx into the cells and de novo synthesis of sphingolipids. Our present findings that ceramide content in media is elevated after palmitate incubation, imply that hepatocytes also secretes newly synthetized ceramide what explains why some researchers did not find ceramide accumulation in liver cells. Importantly, we have shown that CERT, MTP and ABCA1 are the most important proteins which expression was affected by high availability of fatty acids (PA) what probably may be involved in ceramide accumulation and secretion. We observed changes in expression of these transporters which are coincident with ceramide content in hepatocytes and in media what explains the role of CERT, MTP and ABCA1 in accumulation of ceramide in liver cells. Information obtained in this and further studies will be helpful in clarifying the mechanisms of hepatic insulin resistance. Subsequently this knowledge may be used to outline potential therapeutic targets, useful in the treatment of hepatic insulin resistance and type 2 diabetes as well.
